# Mining the pre-diagnostic antibody repertoire of TgMMTV-neu mice to identify autoantibodies useful for the early detection of human breast cancer

**DOI:** 10.1186/1479-5876-12-121

**Published:** 2014-05-10

**Authors:** Jianning Mao, Jon Ladd, Ekram Gad, Lauren Rastetter, Melissa M Johnson, Edmond Marzbani, Jennifer S Childs, Hailing Lu, Yushe Dang, Elizabeth Broussard, Sasha E Stanton, Sam M Hanash, Mary L Disis

**Affiliations:** 1 Tumor Vaccine Group, Center for Translational Medicine in Women’s Health, University of Washington, 850 Republican Street, Box 358050, Seattle, WA 98109, USA; 2 Fred Hutchinson Cancer Research Center, Public Health Science, Arnold Building, M1-B208, PO Box 19024, Seattle, WA 98109, USA; 3 Department of Clinical Cancer Prevention, The University of Texas MD Anderson Cancer Center, 6767 Bertner Street, Unit Number: 1013, Houston, TX 77030, USA

**Keywords:** Cancer diagnostics, Breast cancer, Autoantibodies, Transgenic mice

## Abstract

**Background:**

The use of autoantibodies for the early detection of breast cancer has generated much interest as antibodies can be readily assayed in serum when antigen levels are low. Ideally, diagnostic autoantibodies would be identified in individuals who harbored pre-invasive disease/high risk lesions leading to malignancy. Prospectively collected human serum samples from these individuals are rare and not often available for biomarker discovery. We questioned whether transgenic animals could be used to identify cancer-associated autoantibodies present at the earliest stages of the malignant transformation of breast cancer.

**Methods:**

We collected sera from transgenic mice (TgMMTV-neu) from the time of birth to death by spontaneous mammary tumors. Using sera from a time point prior to the development of tumor, i.e. “pre-diagnostic”, we probed cDNA libraries derived from syngeneic tumors to identify proteins recognized by IgG antibodies. Once antigens were identified, selected proteins were evaluated via protein arrays, for autoantibody responses using plasma from women obtained prior to the development of breast cancer and matched controls. The ability of the antigens to discriminate cases from controls was assessed using receiver-operating-characteristic curve analyses and estimates of the area under the curve.

**Results:**

We identified 6 autoantibodies that were present in mice prior to the development of mammary cancer: Pdhx, Otud6b, Stk39, Zpf238, Lgals8, and Vps35. In rodent validation cohorts, detecting both IgM and IgG antibody responses against a subset of the identified proteins could discriminate pre-diagnostic sera from non-transgenic control sera with an AUC of 0.924. IgG and IgM autoantibodies, specific for a subset of the identified antigens, could discriminate the samples of women who eventually developed breast cancer from case-matched controls who did not develop disease. The discriminatory potential of the pre-diagnostic autoantibodies was enhanced if plasma samples were collected greater than 5 months prior to a breast cancer diagnosis (AUC 0.68; CI 0.565-0.787, p = 0.0025).

**Conclusion:**

Genetically engineered mouse models of cancer may provide a facile discovery tool for identifying autoantibodies useful for human cancer diagnostics.

## Background

A serum test to aid in the diagnosis of cancer has long been the “holy grail” of early cancer detection. Developing a blood based diagnostic assay is ideally suited to a disease such as breast cancer as high risk populations are defined and current screening tools, specifically mammography, are limited in specific populations, i.e. increased breast density in young women. Identification of autoantibodies associated with high risk or pre-invasive disease may facilitate serum-based testing for early detection or risk stratification. Unlike circulating shed tumor proteins or nucleic acids, plasma levels of which are dependent on tumor size, autoantibodies are elicited in significant concentrations even after minimal exposure to the antigen due to cytokine induced production of antibodies by activated B cells [1]. Despite the initial promise of autoantibodies as cancer diagnostic tools, there have been few candidates that demonstrate significant predictive activity in high risk women. Progress has been challenged by a focus on single antibody markers rather than panels, antigen discovery using samples from individuals already bearing invasive cancers, and a lack of understanding of antibody kinetics developing in pre-invasive disease.

Genetically engineered mouse models of cancer may provide a model system for identification of diagnostic autoantibodies. As mice develop spontaneous breast cancers, many in middle age, serum can be collected over time and samples for discovery can span both disease-free and disease-bearing states in the same individual. We questioned whether the TgMMTV-neu model could be used to identify autoantibody candidates for the early detection of human breast cancer. Although the TgMMTV-neu expresses the neu proto-oncogene, the genes expressed in the tumors that arise in these animals is similar to the genes expressed in human luminal breast cancer [2]. The mice have a latency period of pre-invasive disease, sometimes of months, before developing mammary cancers allowing the evolution of an antibody repertoire over time. We utilized serologic screening of pre-diagnostic sera against cDNA libraries of syngeneic tumor expressed in phage to identify antibodies which were present in mice prior to the diagnosis of cancer that could discriminate at risk mice from controls. We then explored whether these autoantibodies had relevance in the early detection of human breast cancer using plasma samples obtained from the Women’s Health Initiative (WHI) study and derived from women who would eventually develop breast cancer as compared to matched controls who remained free of disease.

## Methods

### Murine serum samples

TgMMTV-neu mice (strain name, FVB/N-TgN(MMTV*neu*)-202Mul) or wild-type FVB mice (Jackson Laboratory, San Diego, CA) were bred under specific pathogen-free conditions at the University of Washington. Animal care and use was in accordance with institutional and national guidelines. The protocol was approved by the University of Washington Institutional Animal Care and Use Committee (Protocol number 2878–01). Mice were enrolled for blood collection and monitoring for spontaneous tumor growth between 4–6 weeks of life (Additional file 1: Figure S1A). Blood samples were collected every two weeks by retro-orbital bleeding starting at the time of enrollment until tumor growth dictated euthanasia or approximately 1 year if no tumors developed. Sera was separated and stored in 10 μl aliquots at −80°C until use. Once tumors developed, volume was measured every other day with Vernier calipers and calculated as the product of length × width × height × π/6, or the standard volume calculation for an ellipsoid shape. A colony of FVB wild-type parental animals were also bred and enrolled for blood collection between 4–6 weeks of life following the identical schema (Additional file 1: Figure S1A) as the transgenic mice to provide control age- matched sera.

**Figure 1:**
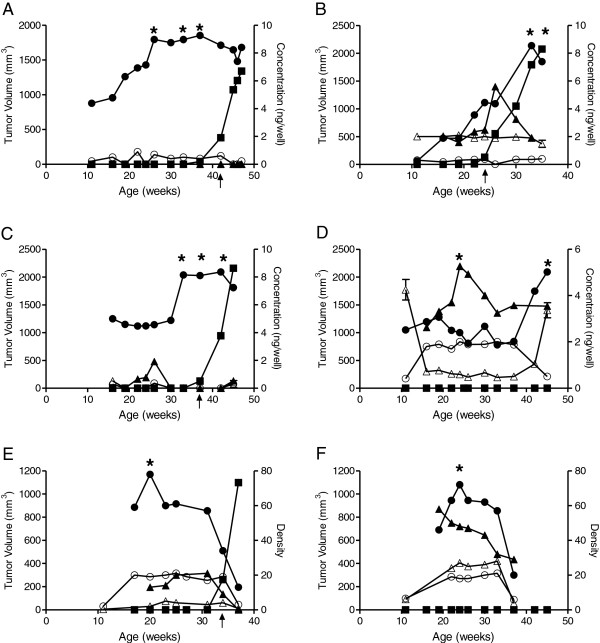
**Tumor associated autoantibodies were detected in the sera of TgMMTV-neu mice prior to the development of palpable disease.** Antigen specific IgG (●), IgM (▲) and tumor growth (■) were identified in a single animal and evaluated over time for **(A)** Pdhx, **(B)** Otud6b, **(C)** Stk39, **(D)** Lgals8, **(E)** Vps35 and **(F)** Znf238. IgG (○) and IgM (∆) measured over time from control animals (n = 20 (+/−SE for first and last time points and for an individual control for all intermediate time points). Left Y axis: Tumor volume; right Y axis: antibody concentration or band density; X axis: mouse age (in weeks). Arrow indicates first palpable tumor. * indicates *p* < 0.05 from initial value.

Serum, taken from animals at time-points approximately 1 month prior to the development of palpable tumor, from 20 individual TgMMTV-neu mice were used for the initial identification of antigens, i.e. pre-diagnostic sera. Pooled serum from 10 FVB wild-type mice of similar age were used as controls in initial SEREX screens as described below (Additional file 1: Figure S1B). Additional single individual FVB wild-type sera were used to define the kinetics of antibody responses (Additional file 1: Figure S1B, Figure 1). For two of the time points, the earliest and latest in each graph, IgG and IgM levels from 20 individual FVB mice are shown (mean and SEM) (Figure 1).

Verification of the antigens was performed using sera derived from an additional 21 TgMMTV-neu mice and an additional 26 FVB age-related wild type mice (Additional file 1: Figure S1C). For verification, all samples were analyzed individually. Samples from the transgenic animals were derived from two time-points in the same individual: approximately 1 month prior to the development of palpable tumors, pre-diagnostic, and the first sample time point after the development of palpable disease, tumor bearing (Additional file 1: Figure S1A). Similar aged samples were analyzed from the parental strain.

### Human plasma samples

De-identified pre-diagnostic plasma samples were collected from 188 women that participated in the WHI Observational Study [3]. These samples consisted of 94 cases (women who eventually developed breast cancer) and 94 controls. Controls were individually matched 1:1 to cases for several variables including age at enrollment (±1 year), race, ethnicity, blood draw date, and clinical center of enrollment. The matching was also performed to ensure that each control had a similar time interval, following her blood draw, as the time from blood draw to breast cancer diagnosis of the case to which she was matched. Samples were stored at -70°C until use. Samples from 48 cases and 48 controls were collected greater than 150 days prior to the cases developing breast cancer and samples from 46 cases and 46 controls were collected within 150 days of the cases being diagnosed with breast cancer.

### Identification of tumor antigens

Serological screening of cDNA expression libraries (SEREX) was used to identify tumor associated autoantibodies as previously described [4]. Briefly, a cDNA expression library was constructed from a syngeneic tumor cell line [4]. Pre-diagnostic serum samples pooled from 20 individual animals were used for the identification of autoantibodies. A total of 3 × 10^6^ recombinant clones per library were screened. A median of 4 (range 0–8) discrete positive plaques were identified from each group of 32,000 clones evaluated. The positive plaques were interrogated with pooled normal FVB serum (Additional file 1: Figure S1B). Clones that did not react to the FVB control serum were then purified to moncolonality and converted to pBluescript phagemid and the nucleotide sequences of the cDNA inserts were determined using an ABI Prism automated DNA sequencer. Blast was used to assess sequence homology. Six unique proteins were found to elicit autoantibodies in the pre-diagnostic sera of TgMMTV-neu mice and not the FVB parental serum pool (Table 1).

**Table 1:** Pre-diagnostic antigens identified using SEREX

**Genes**	**Full gene name**	**SubcellularLocation**	**Homology**
Phdx	Pyruvate dehydrogenase complex	Mitochondria	89%
Otud6b	OTU domain containing 6B	Cytoplasm and Nucleus	95%
Stk39	Serine/threonine kinase 39 (STE20/SPS1 homolog, yeast)	Cytoplasm and Nucleus	94%
Zfp238	Zinc finger protein 238	Nucleus	100%
Lgals8	Lectin, galactoside-binging, soluble,8	Cytoplasm	80%
Vps35	Vacuolar protein sorting 35	Cytoplasm	99%

Evaluation of autoantibodies in mice. Two methods were used for the quantitation of murine antibodies; ELISA and western blot/densitometry. The measurement of serum antibodies to tumor antigen Pdhx was carried out using a lysate ELISA (bacteria expressing the protein of interest) as previously described with the modification that both IgG and IgM responses were evaluated (Lu et al. Cancer Research 2006). Experimental serum at a 1:200 dilution and horseradish peroxidase conjugated goat anti-mouse IgG (diluted 1:5,000) or IgM (diluted 1:1000; Zymed, San Francisco, CA) were used. After development, plates were read at an absorbance of 450 nm. The OD of each serum dilution was calculated as the OD of the antigen-coated wells minus the OD of non-antigen encoding lysate-coated wells. The autoantibody concentration (ng/well) was calculated from the 4-parameter fitted standard curve on each plate. All identified antigens were highly homologous between mouse and man, median 95% (range 80–100). Human recombinant proteins were available for Otud6b, Stk39, and Lgals8 (all from Abnova, Walnut CA) and indirect ELISA was performed to measure serum antibodies to tumor antigens as previously described [5]. Experimental serum at a 1:200 dilution and goat anti-mouse IgG antibody (1:100,000 dilution) or goat anti-mouse IgM antibody (1:5,000 dilution) (Invitrogen, Grand Island, NY) were used. Plates were read at 450 nm. The autoantibody concentration (ng/well) was calculated from the 4-parameter fitted standard curve on each plate. Five positive and negative samples were analyzed by western blot for each antigen with each assay demonstrating a sensitivity and specificity greater than 75%.

The assessment of Vps35 and Znf238 autoantibodies was performed by Western blot and densitometry as the recombinant proteins gave insufficient signal in ELISA. 3 pmole recombinant protein of Vps35 or Znf238 (Abnova) were loaded on SDS-PAGE gel and transferred onto nitrocellulose membranes (Amersham Pharmacia Biotech, Piscataway, NJ). After blocking with 5% nonfat milk, membranes were incubated with mouse sera (diluted 1:100) overnight. Then the membranes were washed with TBS/0.05% Tween 20 and incubated with peroxidase-labeled goat anti-mouse IgG (1:1,000 dilution) or IgM (1:500 dilution) secondary antibody (Invitrogen). After washing, bands were visualized using a peroxidase-linked enhanced chemiluminescence detection system (Amersham Pharmacia Biotech) and the optical density of the specific band was quantified with ImageJ software.

Evaluation of antigen expression in human DCIS. To evaluate whether these identified antigens had the potential for being expressed in human pre-invasive lesions, a GEO dataset, GSE26304, was analyzed for expression of the genes encoding the identified pre-diagnostic antigens [6]. This published gene expression data was derived from 31 ductal carcinoma in situ (DCIS) and 36 invasive ductal carcinomas (IDC). Six normal breast tissue samples were included as controls. Data for the pre-diagnostic antigens are expressed as normalized to beta actin. The mean value and 2 standard deviations of the expression of a particular antigen in normal breast tissue was calculated to determine the incidence of DCIS or IDC samples having higher expression of the candidate antigens compared to normal breast tissue.

### Evaluation of pre-diagnostic autoantibodies in women without cancer

Due to the low volume of sample available for analysis, serum autoantibodies to only 3 antigens, Otud6B, Stk39, and Pdhx, were assessed using customized protein microarrays as previously described [7]. We chose these proteins as, in mice, antibodies to these 3 antigens were significantly elevated at multiple time points prior to the development of clinically palpable cancer (Figure 1). In brief, recombinant proteins were arrayed in duplicate onto nitrocellulose-coated slides using a contact printer. Plasma samples (diluted 1:150) were hybridized with the protein microarray for 3 hours at 4°C. Slides were then incubated with Cy5-labeled anti-human IgG for 1 hour at 4°C, followed by incubation with Cy3-labeled anti-human IgM for 1 hour at 4°C. Local background-subtracted median spot intensities for downstream statistical analysis of both IgG and IgM were generated using GenePix software.

### Statistical analysis

For all analysis, significance was assessed using a two-tailed T test or ANOVA with p < 0.05 being considered significant. Differences in the incidence of an antibody response between transgenic mice and controls were evaluated using a x^2^ test. The sensitivity and specificity of a single or combination of antibodies in mice was evaluated using receiver-operating-characteristic (ROC) curve analyses, leading to estimates of the area under the curve (AUC), with 95% confidence intervals. Statistical analysis was carried out in SPSS software, version 15.0. For protein arrays, ROC analysis of marker combinations was performed using a linear regression model based on maximum likelihood estimation. AUC and 95% confidence intervals were calculated using R v2.13.1.

## Results

### Tumor associated autoantibodies were detected in the sera of TgMMTV-neu mice prior to the development of palpable disease

Pre-diagnostic autoantibodies directed against six tumor associated antigens were identified in individual TgMMTV-neu mice at significantly higher levels compared to wild-type controls (p < 0.01 for each antigen) (Table 1, Figure 1). The antigens are all intracellular proteins of diverse functions and are involved in glycolysis, signaling, and cell adhesion pathways. Half of the antigens (Pdhx, Lgals8, and Otud6) have also been associated with inflammation and/or autoimmunity. All are highly homologous to a corresponding human protein (Table 1). Tumor growth and antibody kinetics are shown for the individual animals in which the specific antibody was detected (Figure 1). In all individuals, antigen specific IgG antibodies significantly increased over time (p < 0.05 compared to baseline) prior to tumor detection (Figure 1, A-F). Autoantibodies specific for Lgals8 and Znf238 were discovered in mice that did not develop tumor during the time course of the study (Figure 1, D, E). IgM antibodies were also detected prior to palpable breast tumors in most animals, in some cases at much higher levels than IgG (Figure 1B, D-F). In general, levels of IgM antibodies decreased over time, while IgG antibodies either increased or persisted at measurable levels after tumor development.

### Both IgG and IgM responses are needed to discriminate serum derived from mice destined to develop tumor as compared to controls

**Figure 2:**
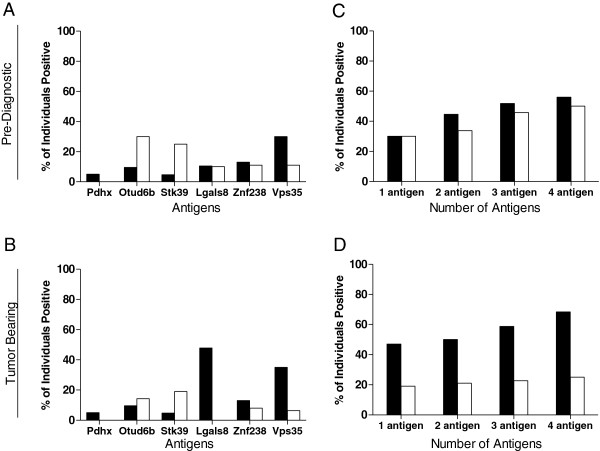
**IgM antibodies to pre-diagnostic antigens are more commonly identified in sera from mice prior to the development of palpable breast cancer.** Incidence of IgG (black bar) and IgM (white bar) for antigen Pdhx, Otud6b, Stk39, Lgals8, Znf238 and Vps35 in sera obtained **(A)** pre-diagnosis or when **(B)** tumor bearing. Incidence of IgG (black bar) and IgM (white bar) antibody detection for a panel with any 1 antigen, 2 antigens, 3 antigens and 4 antigens in sera obtained **(C)** pre-diagnosis or when **(D)** tumor bearing.

We further evaluated the prevalence of the pre-diagnostic autoantibodies in serum samples taken from an additional 21 transgenic animals not used for the autoantibody discovery. The incidence of autoantibodies to an individual antigen in mice, both pre and post tumor development, is shown in Figure 2. The incidence of response to the antigens, prior to the development of palpable tumor, ranged from 5% (Pdhx) to 30% of mice for Vps35 for IgG. For IgM, prior to tumor detection, incidence of autoantibody responses ranged from 0% (Pdhx) to 30% (Otud6b) (Figure 2A). In tumor bearing mice, IgG autoantibody incidence ranged from 5% (Pdhx, Stk39) to 50% (Lgals8). IgM responses were found in 0% (Pdhx, Lgals8) to 20% (Stk39) of tumor bearing mice (Figure 2B). The incidence of IgG or IgM autoantibodies to any of the identified antigens was less than 10% of the 20 control mice, with the exception of Znf238 (22% for IgG). While IgG antibodies to any of the antigens could be detected in 68% of tumor bearing samples, IgM responses to the same proteins were present in only 25% (p < 0.05) (Figure 2D). In pre-diagnostic sera, IgM antibodies could be detected in over half the mice at levels similar to the antigen specific IgG antibodies (Figure 2C). For a panel of all 4 markers, 56% of pre-diagnostic sera contained IgG antibodies to any of the antigens and 50% contained IgM. No individual responded to more than 4 of the antigens.

To assess the potential utility of the autoantibodies in discriminating those animals that would develop mammary tumors (pre-diagnostic and tumor bearing samples) from FVB controls, ROC were generated and AUC calculated (Table 2). The performance of IgG antibodies was equivalent in discriminating both case sample sets from controls with a panel of 3 markers demonstrating superior performance. A combination of Otud6b, Stk39 and Lgals8 gave an AUC of 0.868 (95% CI 0.744-0.968, p < 0.001) for pre-diagnostic sera and 0.871 for sera derived from tumor bearing mice (95% CI 0.744-0.976, p < 0.001) with a sensitivity of 0.75 and specificity of 0.8. The performance of the 3 antigen panel for IgM was superior in the pre-diagnostic sera with an AUC of 0.841 (95% CI 0.7-0.966, p < 0.001) with a sensitivity of 0.7 and specificity of 0.9 as compared to tumor bearing sera, at 0.676 (95% CI 0.48-0.84, p = 0.141). A combination of IgG and IgM was most effective in modeling early detection with an AUC of 0.924 (95% CI 0.81-1.0, p < 0.001) with a sensitivity of 0.85 and specificity of 0.9 discriminating pre-diagnostic sera from FVB controls. Once animals had evidence of palpable tumor, the AUC decreased to 0.676 (95% CI 0.48-0.84, p = 0.141) (Table 2). All AUC for the various combinations are shown in Additional file 1: Table S1.

**Table 2:** AUC of IgG and IgM antibodies against pre-diagnostic antigens

**Antigens**	**IgG**		**IgM**		**IgG + IgM**	
	**Pre-diagnostic**	**Tumor bearing**	**Pre-diagnostic**	**Tumor bearing**	**Pre-diagnostic**	**Tumor bearing**
Phdx	0.507	0.507	0.500	0.500	0.507	0.507
Otud6b	0.784	0.784	0.782	0.574	0.818	0.658
Stk39	0.640	0.640	0.582	0.547	0.704	0.591
Lgals8	0.781	0.813	0.732	0.574	0.737	0.593
Vps35	0.500	0.500	0.503	0.500	0.508	0.500
Znf238	0.522	0.548	0.661	0.541	0.648	0.534
Otud6b + Stk39 + Lgals8	0.868	0.871	0.841	0.676	0.924	0.676

### Pre-diagnostic tumor antigens identified in mice may be useful for the early detection of human breast cancer

**Figure 3:**
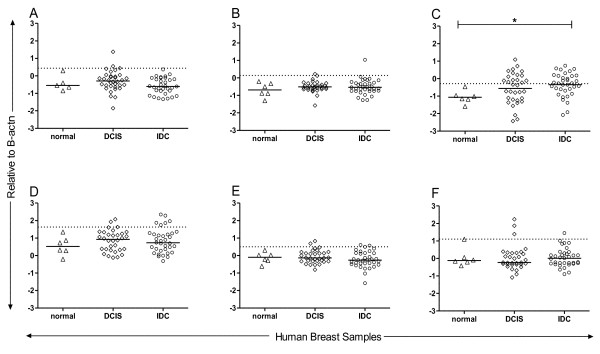
**Pre-diagnostic autoantibodies target proteins that may be expressed in pre-invasive breast lesions.** Gene expression relative to beta actin for Pdhx **(A)**, Otud6b **(B)**, Stk39 **(C)**, Lgals8 **(D)**, Vps35 **(E)** and Znf238 **(F)** in normal breast (n = 6), DCIS (n = 31) and IDC (n = 36). Dashed line; mean + 2SD of normal breast tissue for each antigen. * indicates *p* < 0.05.

To explore the relevance of these antigens to human pre-invasive disease, we evaluated gene expression in an existing data set of normal breast, DCIS, and IDC. 10% of DCIS expressed Pdhx (Figure 3A), Lgals8 (Figure 3D) and Znf238 (Figure 3F) at levels greater than 2 standard deviations above normal breast tissues. Lgals8 (Figure 3D) was also expressed above normal levels in 17% of IDC. Stk39 (Figure 3C) gene expression was upregulated in 50% of both DCIS and IDC compared to normal breast tissue (p < 0.05). Otud6b and Vps35 expression was similar across all samples (Figure 3B, E).

As human homologues of the murine pre-diagnostic antigens exist and the proteins appear to be expressed in human pre-invasive breast lesions, we explored the potential utility of three of the antigens, Pdhx, Otud6B, and Stk39 in discriminating women who would eventually develop cancer from controls (Table 3). Limited volumes of samples available limited our exploration to 3 of the 6 identified proteins. Otud6B and Stk39 were chosen as part of the panel of antigens that demonstrated superior performance in the mice. Pdhx was added as this antigen was identified in mice that eventually developed mammary cancer (unlike Lgals8) and Pdhx autoantibodies were significantly elevated at the earliest time points tested in the mice (Figure 1A). An evaluation of the population as a whole revealed a range of AUC for IgG and IgM responses from 0.52-0.56 for each individual antigen. A panel of all three gave an AUC of 0.59 (95% CI 0.512-0.675, p = 0.026) for IgG, 0.56 (95% CI 0.481-0.645, p = 0.135) for IgM and the combination of IgG and IgM, 0.59 (95% CI 0.511-0.674, p = 0.028).

**Table 3:** WHI sample characteristics

		**Less than 150 Days**		**Greater than 150 Days**	
		**Cases**	**Controls**	**Cases**	**Controls**
Number		48	48	46	46
Age		64.6 (51 – 78)	64.5 (51 – 78)	64.2 (50 – 77)	64.3 (50 – 77)
Stage	I	-	-	-	-
	II	37	-	35	-
	III	10	-	11	-
	IV	1	-	-	-
Days prior to diagnosis		82.4 (12–148)	-	209.2 (151 – 264)	-

Kinetic studies in mice (Figure 1) demonstrated significant fluctuation in IgM antibody levels in time periods distant to tumor detection. We, therefore, evaluated the performance of the antibody panel on the pre-diagnostic human samples separated by the case median time-from-diagnosis (150 days) (Table 4). A linear regression analysis of IgG responses demonstrated more discrimination between case and control for samples collected greater than 150 days prior to diagnosis, AUC = 0.62 (95% CI 0.506-0.735, p = 0.043), than those collected closer to diagnosis, AUC = 0.59 (95% CI 0.476-0.707, p = 0.123). A combination of the 3 antigens for IgM responses were equivalent for samples collected both further from diagnosis, AUC = 0.61 (95% CI 0.493-0.727, p = 0.068), and those collected closer to diagnosis, AUC = 0.61 (95% CI 0.495-0.722, p = 0.064). A combination of both Ig isotypes increased the AUC of both groups, but was superior for the samples collected greater than 150 days prior to diagnosis, AUC 0.68 (CI 0.565-0.787, p = 0.003). The sensitivity and specificity of the IgG + IgM panel in discriminating women greater than 150 days from their breast cancer diagnosis from control is 67% and 65% respectively. We had previously screened the WHI pre-diagnostic sera for antibodies to HER2, p53 and cyclin B1 using the same approach [8]. Combination of IgG and IgM to all 6 antigens further increased the AUC to 0.75 in samples collected greater than 150 days from diagnosis.

**Table 4:** AUC of IgG and IgM antibodies against pre-diagnostic antigens based on time to diagnosis

**Antigens**	**Greater than 150 days**			**Less than 150 days**		
	**IgG**	**IgM**	**IgG + IgM**	**IgG**	**IgM**	**IgG + IgM**
Phdx	0.62	0.56	0.62	0.52	0.56	0.57
Otud6b	0.53	0.57	0.58	0.58	0.48	0.58
Stk39	0.51	0.50	0.49	0.52	0.60	0.60
All 3	0.62	0.61	0.68	0.59	0.61	0.63

## Discussion

We questioned whether the use of genetically engineered mouse models of mammary cancer could serve as a tool for developing autoantibody panels useful for the early detection of human breast cancer and/or improved stratification of risk. The identification of cancer predicting autoantibodies, using individuals bearing early stage invasive breast cancers, may not encompass antigens associated with the earliest high risk lesions, thus may limit the utility of any identified panel for screening a general population. Transgenic mouse models of mammary cancer have been shown to have significant genetic similarities to human breast cancer [2]. In addition, the serum autoantibody repertoire induced in mouse and man by breast cancer is also similar [4]. Using SEREX, and screening tumor cDNA libraries expressed in phage with sera from cancer bearing mice, a tumor associated autoantibody repertoire could be identified for the TgMMTV-neu. Nearly half the identified antigens were reported as human tumor antigens in a variety of cancers. Data presented here demonstrate that a pre-diagnostic autoantibody repertoire can be identified in mice prior to the development of spontaneous tumors and these antibodies may be useful for the detection of human breast cancer. Further, our work shows that detection with a combination of both IgG and IgM antibodies for a specific antigen may improve the ability to identify patients harboring the disease at time points more distant from diagnosis than is achievable by the use of IgG autoantibodies alone.

There have been several studies that have explored the use of autoantibody panels in the early detection of breast cancer. All have assessed sera from disease bearing individuals and several have focused on well-known tumor associated antibodies such as HER2, p53, NY-ESO, and MUC1 for example [8,9]. In an evaluation of 94 patients with breast cancer and 40 patients with DCIS, the percentage of patients with detectable autoantibodies specific for a panel of 7 known tumor associated antigens, ranged from 55-73% in patients with invasive tumors but only 20-62% in women with DCIS (based on responses in volunteer controls) [9]. A variety of investigations have evaluated array-based approaches in identifying autoantibodies that are associated with discriminating DCIS from invasive breast cancer [10-12]. Almost all the autoantibodies identified were intracellular proteins. The panels could discern DCIS from invasive cancers with AUCs of approximately 0.7-0.8 although none have been explored in high risk women. All the identified panels contained proteins that could be implicated in cancer biology and pathogenesis [11].

The autoantibodies we found in the pre-diagnostic sera of TgMMTV-neu mice, animals that had not yet developed invasive cancers, were also directed against intracellular proteins. Half of the antigens identified are also associated with inflammation and immunity. Pdhx, a glycolysis protein, is an antigenic component of anti-mitochondrial autoantibodies. The majority of patients with primary billiary cirrhosis have autoantibodies directed against Pdhx [13]. Lgals8 is a cytosolic lectin which has recently been shown to bind to damaged host glycans and stimulate autophagy [14]. Antibodies to Lgals8 have been identified in a variety of autoimmune diseases including systemic lupus erythematosis and rheumatoid arthritis and may play a role in regulating autoimmune inflammation [15,16]. Otud6b is a protease that cleaves ubiquitin linkages and its expression has recently been shown to be involved in the regulation of B cell proliferation after cytokine stimulation [17]. The remaining antigens have no link to inflammation. Stk39 functions in the cellular stress pathway and activates p38 MAP kinase. Stk39 is involved in cation-chloride transport and polymorphisms in this gene are associated with the development of hypertension [18]. Zfp238, a transcription repressor protein has been shown to be necessary for neuronal survival and development [19]. Vps35 is part of a larger complex involved in retrograde transport of proteins from endosomes to Golgi. Mutations in Vps35 have been associated with the development of Parkinson’s disease [20]. None of the proteins has been shown to play a major role in breast cancer initiation or progression. Inflammation has long been associated with cancer initiation due to the proliferative environment induced by innate immune cells and B cells [21]. The presence of inflammation associated and autoimmune related antibodies in our panel may be reflective of alterations occurring at the earliest stages of the malignant transformation. However, as these autoantibodies may also be elevated in women with chronic inflammatory and autoimmune disease, and their potential clinical utility for screening might be limited in this population.

Genes encoding homologues of murine pre-diagnostic antigens are found in human breast, DCIS, and invasive cancers. Our studies demonstrate that women who eventually develop breast cancer have evidence of autoantibodies targeting these pre-diagnostic antigens prior to their first diagnosis of disease. A limited panel of only 3 of the autoantibodies could discern women destined to develop breast cancer from matched controls that did not develop disease with an AUC approaching 0.7. Key to this finding was the use of both IgG and IgM antibodies for detection and assessing sera at a time point more distant from diagnosis. IgM antibodies are the first antibodies secreted by B cells in response to an antigen. B cell proliferation and IgM production are induced simultaneously via cytokine secretion by a number of different immune cells [22]. As T cells become involved in antigen recognition, immunoglobulin class switching occurs with IgG responses becoming predominant and persistent and IgM antibodies wane. Indeed, several lines of evidence suggest that the presence of IgM antibodies will increase the secretion of IgG antibodies to greater levels than those achieved if IgM antibodies are not present [22]. The kinetics of both the IgG and IgM antibody response in the TgMMTV-neu mice (Figure 1) demonstrate that antigen specific IgM levels may be quite elevated in the time period distant from diagnosis, but decrease as IgG antibodies become elevated, similar to what is observed in viral infections. The ability to detect both IgG and IgM autoantibodies appears to provide broader population coverage in the pre-diagnostic setting in the mice and our data in women would indicate the same. A yearly screening strategy, for example, may capture a time point where both IgM and IgG antibodies are elevated potentially increasing the sensitivity of serologic screening with autoantigens.

The ideal method to develop an autoantibody panel for the early detection of breast cancer would be to discover autoantigens in women who have not yet developed invasive cancer but are destined to do so. The use of genetically engineered mice that develop spontaneous tumors, allows investigators to model studies such as WHI and collect samples from individuals who have not yet developed cancer but will do so in a defined period of time. Genetic and protein based analyses suggest that there is enough similarity between cancers arising in mice and people that discovery methods in mice might be applicable to human cancer diagnostics.

## Conclusions

Data presented in this report suggest that, at least for breast cancer, mining the pre-diagnostic autoantibody repertoire of transgenic mice yields viable biomarker candidates for the early detection of the disease.

## Abbreviations

AUC: Area under the curve; DCIS: Ductal carcinoma in situ; IDC: Invasive ductal carcinomas; ROC: Receiver operating curves; SEREX: Serological screening of cDNA expression libraires; WHI: Womens Health Initiative.

## Competing interests

MLD and SH are inventors on patents held by academic institutions that pertain to data presented in this manuscript.

## Authors’ contributions

JM and JL executed the experiments as described. EG and LR developed the sera repository for the mice. MMJ, EM, YD, HL, EB, SES contributed laboratory and data analysis support for the experiments described. JSC provided regulatory support. SH and MLD supervised all studies and contributed to data interpretation and manuscript writing and review. All authors read and approved the final manuscript.

## Supplementary Material

Additional file 1 Serum acquisition and mouse cohorts. Click here for file
