# Supplementary material for: Mining the pre-diagnostic antibody repertoire of TgMMTV-neu mice to identify autoantibodies useful for the early detection of human breast cancer
Source: J Transl Med. 2014 May 10;12:121. doi: 10.1186/1479-5876-12-121 (PMC4022541; doi:10.1186/1479-5876-12-121)
Supplement: Additional file 1 — Serum acquisition and mouse cohorts. [file 1479-5876-12-121-S1.pdf]

Supplemental Table 1. AUC to the combinations of discovered antigens for pre-diagnostic (non-palpable) and tumor bearing (palpable) serum samples (n=21) as compared to FVB parental controls (n=20)

Antigens	IgG		IgM		IgG+IgM	
	non-palpable	palpable	non-palpable	palpable	non-palpable	palpable
Phdx	0.507	0.507	0.5	0.5	0.507	0.507
Otud6b	0.784	0.784	0.782	0.574	0.818	0.658
Stk39	0.64	0.64	0.582	0.547	0.704	0.591
Lgals8	0.781	0.813	0.732	0.574	0.737	0.593
Vps35	0.5	0.5	0.503	0.5	0.508	0.5
Znf238	0.522	0.548	0.661	0.541	0.648	0.534
Phdx+Otud6b	0.751	0.751	0.681	0.512	0.751	0.62
Pdhx+Stk39	0.644	0.644	0.512	0.52	0.691	0.63
Pdhx+Lgals8	0.747	0.809	0.714	0.54	0.712	0.562
Pdhx+Vps35	0.487	0.487	0.5	0.5	0.497	0.487
Pdhx+Znf238	0.512	0.597	0.64	0.52	0.64	0.522
Otud6b+Stk39	0.85	0.85	0.798	0.621	0.871	0.738
Otud6b+Lgals8	0.853	0.87	0.773	0.647	0.791	0.711
Otud6b+Vps35	0.744	0.744	0.712	0.487	0.784	0.548
Otud6b+Znf238	0.714	0.732	0.784	0.528	0.798	0.61
Stk39+Lgals8	0.774	0.804	0.749	0.615	0.788	0.689
Stk39+Vps35	0.62	0.62	0.524	0.492	0.64	0.549
Stk39+Znf238	0.614	0.634	0.673	0.584	0.712	0.64
Lgals8+Vps35	0.712	0.74	0.716	0.52	0.687	0.512
Lgals8+Znf238	0.74	0.734	0.687	0.54	0.696	0.52
Vps35+Znf238	0.476	0.454	0.62	0.512	0.618	0.496
Phdx+Otud6b+Stk39	0.814	0.814	0.774	0.614	0.87	0.712
Pdhx+Otud6b+Lgals8	0.812	0.844	0.712	0.644	0.712	0.687
Phdx+Otud6b+Vps35	0.74	0.712	0.674	0.49	0.744	0.52
Pdhx+Otud6b+Znf238	0.71	0.712	0.76	0.518	0.787	0.598
Pdhx+Stk39+Lgals8	0.74	0.812	0.724	0.62	0.78	0.664
Pdhx+Stk39+Vps35	0.614	0.614	0.52	0.486	0.622	0.564
Pdhx+Stk39+Znf238	0.64	0.624	0.664	0.558	0.72	0.664
Pdhx+Lgals8+Vps35	0.667	0.71	0.683	0.516	0.664	0.557
Pdhx+Lgals8+Znf238	0.72	0.72	0.614	0.514	0.698	0.534
Pdhx+Vps35+Znf238	0.498	0.476	0.614	0.558	0.653	0.518
Otud6b+Stk39+Lgals8	0.868	0.871	0.841	0.676	0.924	0.676
Otud6b+Stk39+Vps35	0.815	0.776	0.744	0.624	0.776	0.714
Otud6b+Stk39+Znf238	0.814	0.786	0.814	0.614	0.844	0.712
Otud6b+Lgals8+Vps35	0.824	0.84	0.714	0.626	0.786	0.696
Otud6b+Lgals8+Znf238	0.814	0.865	0.754	0.612	0.818	0.676
Otud6b+Vps35+Znf238	0.74	0.726	0.716	0.498	0.653	0.616
Stk39+Lgals8+Vps35	0.732	0.767	0.714	0.598	0.746	0.614
Stk39+Lgals8+Znf238	0.726	0.764	0.754	0.578	0.784	0.692
Stk39+Vps35+Znf238	0.578	0.564	0.534	0.512	0.683	0.534
Lgals8+Vps35+Znf238	0.746	0.734	0.664	0.512	0.678	0.523

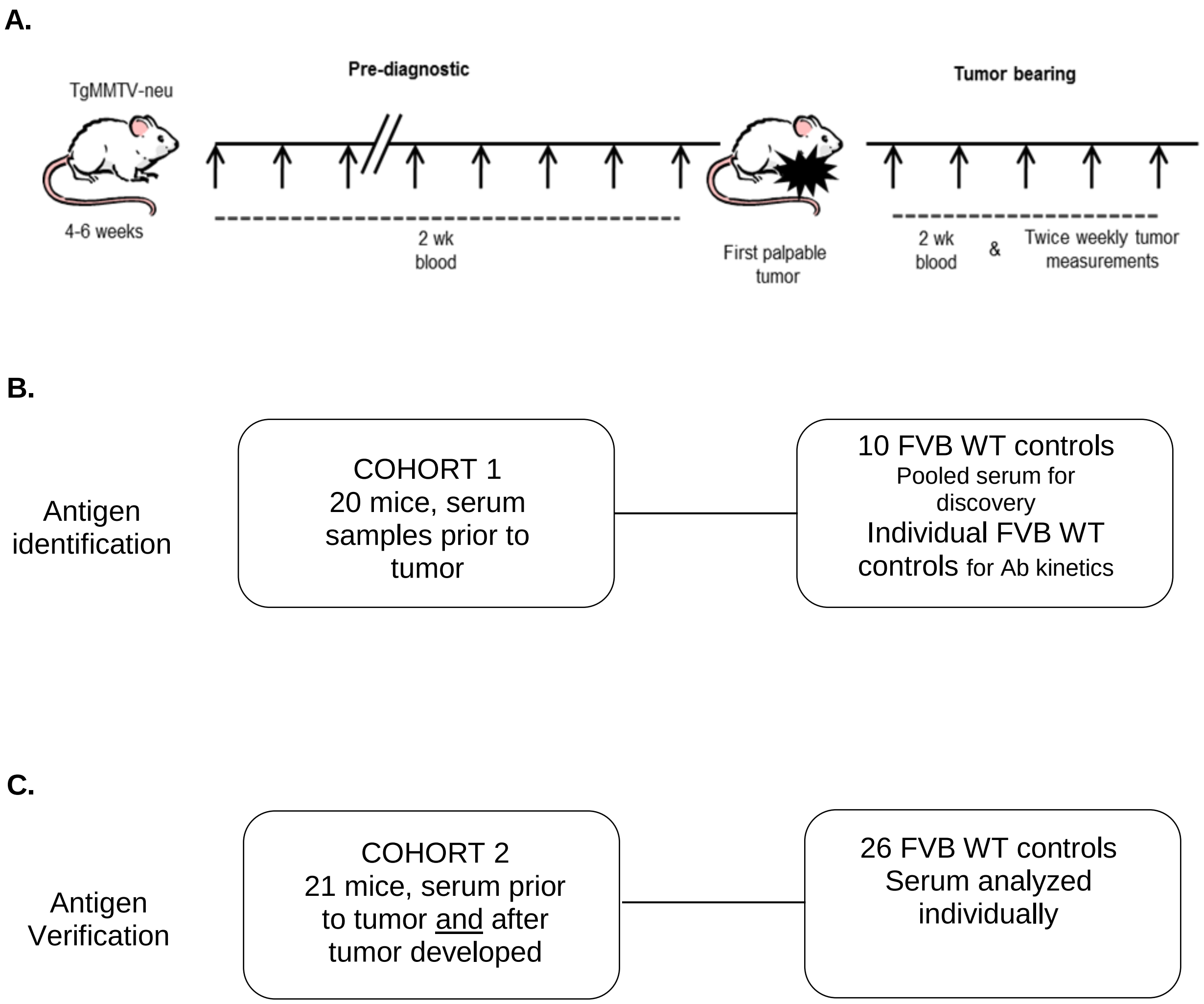

Supplemental Figure 1
